# An Ecologic Analysis of County-Level PM_2.5_ Concentrations and Lung Cancer Incidence and Mortality

**DOI:** 10.3390/ijerph8061865

**Published:** 2011-06-01

**Authors:** Lisa C. Vinikoor-Imler, J. Allen Davis, Thomas J. Luben

**Affiliations:** National Center for Environmental Assessment, U.S. Environmental Protection Agency, 109 T.W. Alexander Dr, MD B243-01, Research Triangle Park, NC 27711, USA; E-Mails: davis.allen@epa.gov (J.A.D.); luben.tom@epa.gov (T.J.L.)

**Keywords:** particulate matter, lung cancer, incidence rates, mortality rates

## Abstract

Few studies have explored the relationship between PM_2.5_ and lung cancer incidence. Although results are mixed, some studies have demonstrated a positive relationship between PM_2.5_ and lung cancer mortality. Using an ecologic study design, we examined the county-level associations between PM_2.5_ concentrations (2002–2005) and lung cancer incidence and mortality in North Carolina (2002–2006). Positive trends were observed between PM_2.5_ concentrations and lung cancer incidence and mortality; however, the R^2^ for both were <0.10. The slopes for the relationship between PM_2.5_ and lung cancer incidence and mortality were 1.26 (95% CI 0.31, 2.21, p-value 0.01) and 0.73 (95% CI 0.09, 1.36, p-value 0.03) per 1 μg/m^3^ PM_2.5_, respectively. These associations were slightly strengthened with the inclusion of variables representing socioeconomic status and smoking. Although variability is high, thus reflecting the importance of tobacco smoking and other etiologic agents that influence lung cancer incidence and mortality besides PM_2.5_, a positive trend is observed between PM_2.5_ and lung cancer incidence and mortality. This suggests the possibility of an association between PM_2.5_ concentrations and lung cancer incidence and mortality.

## 1. Introduction

Although results have been mixed, overall, recent studies have reported an association between PM_2.5_ exposure and lung cancer mortality [1–8]. However, few studies have been conducted to examine the association between PM_2.5_ exposure and lung cancer incidence. Analyses performed on the Netherlands Cohort Study on Diet and Cancer (NCLS) reported no associations between PM_2.5_ and lung cancer incidence [1]. In addition, two recent studies examined the association between PM_10_ and lung cancer incidence [9,10]. One study conducted in Europe reported no association [10], whereas the other study did find a positive relationship between PM_10_ and lung cancer incidence among men in California [9]. The objective of this study was to further investigate the association between PM_2.5_ and lung cancer incidence rates, as well as, lung cancer mortality rates, using an ecologic study conducted across North Carolina. This will provide information on the possibility of an association between PM_2.5_ and lung cancer incidence, an association rarely examined.

## 2. Methods

For this ecologic study, age-adjusted lung cancer incidence and mortality rates from 2002–2006 were obtained for each county in North Carolina (n = 100) from the North Carolina Central Cancer Registry and the State Center for Health Statistics. Predictions of the PM_2.5_ concentrations for the years 2002 through 2005 were determined using a hierarchical Bayesian model that combined monitoring data from the U.S. Environmental Protection Agency’s (EPA) Air Quality System (AQS) with numerical output from the EPA’s Community Multi-Scale Air Quality (CMAQ) model [11]. The model provides information on areas of the state without monitors by modeling known emissions and meteorological conditions. This is combined with actual monitoring data where available. This method has been validated and provides reliable information on PM_2.5_ [11]. The estimated PM_2.5_ concentrations were reported for a grid consisting of 12 × 12 km cells spanning the spatial extent of North Carolina. We calculated the 24-hour average PM_2.5_ values for each grid cell during the 2002 to 2005 time period. The average across this time period was then calculated for each county using spatial weighting to account for counties that spanned multiple grid cells. The weighting was performed by having each grid cell contribute to the county-wide estimate based on the proportion of the county’s area in each cell.

Linear regression was performed in order to estimate the relationship between PM_2.5_ concentration and lung cancer incidence and mortality rates. To adjust for neighborhood socioeconomic status (SES) and smoking, the analyses were repeated with a variable for each included in the models. County-level per capita income estimates were obtained from the North Carolina Comprehensive Assessment for Tracking Community Health (CATCH) system of the State Center for Health Statistics. Smoking statistics were not available for each individual county. However, cigarette smoking prevalence rates from the 2005 North Carolina Behavioral Risk Factor Surveillance System (BRFSS) were available for some single counties (n = 22) and some combined counties (n = 78; 13 groups ranging from 3 to 15 counties with a median of 5 counties per group). Rates for the combined counties were applied to all counties in each grouping.

Linear regression was also used to evaluate if associations exist between PM_2.5_ concentrations and breast and prostate cancers rates from the North Carolina Central Cancer Registry and the State Center for Health Statistics. This additional analysis was undertaken to examine if an association would be prevalent solely for lung cancer or for multiple cancers, including those not thought to be related to air pollutants.

## 3. Results

**Figure 1.**
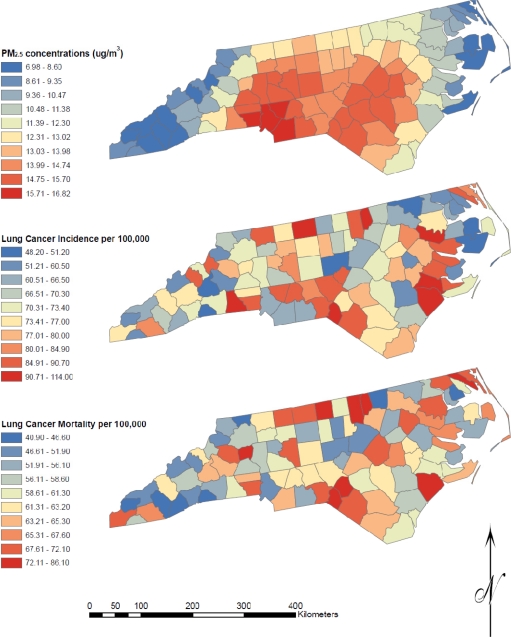
Maps of PM_2.5_ concentrations, lung cancer incidence, and lung cancer mortality for the 100 counties in North Carolina.

Age-adjusted lung cancer incidence and mortality rates, as well as average PM_2.5_ concentrations, are displayed on maps of the 100 counties in Figure 1.

County-wide age-adjusted lung cancer incidence rates for 2002–2006 ranged from 48.2–114.0 per 100,000, with a median of 72.9 per 100,000. For lung cancer mortality, the median rate was 61.3 per 100,000 with a range of 40.9–86.1 per 100,000. The four year average of 24-hour PM_2.5_ concentrations ranged from 7.0 μg/m^3^ for the county with the lowest concentration to 16.8 μg/m^3^ for the county with the highest concentration. The highest PM_2.5_ concentrations were found in Mecklenberg county, where Charlotte (the largest urban center in NC) is located, and the surrounding counties. The lowest PM_2.5_ concentrations were found in counties on the eastern coast and western border of the state.

We observed high variability between county-averaged PM_2.5_ concentrations and both lung cancer incidence and mortality rates across the 100 counties in North Carolina. R^2^ values were 0.07 for the association between PM_2.5_ concentration and lung cancer incidence rates and 0.05 for the association between PM_2.5_ concentration and lung cancer mortality rates.

**Figure 2.**
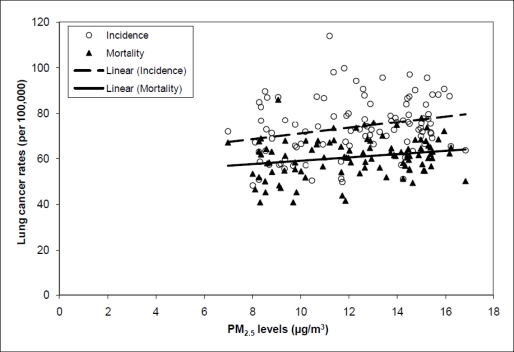
Lung Cancer Incidence and Mortality Rates *versus* PM_2.5_ levels for 100 counties in North Carolina.* * Unadjusted linear models. Slopes: Incidence 1.26 per 1 μg/m^3^ PM_2.5_ (p-value 0.01) and Mortality 0.73 per 1 μg/m^3^ PM_2.5_ (p-values 0.03)

Despite the low R^2^ values, an overall trend was observed, with counties that had higher levels of PM_2.5_ also having higher lung cancer incidence and mortality rates (Figure 2), although there are exceptions, such as Mecklenberg county. The unadjusted slope for the linear trend between PM_2.5_ concentrations and lung cancer incidence was 1.26 (95% CI 0.31, 2.21, p-value 0.01) per 1 μg/m^3^ PM_2.5_. For lung cancer mortality, the slope of the relationship with PM_2.5_ concentration was 0.73 (95% CI 0.09, 1.36, p-value 0.03) per 1 μg/m^3^ PM_2.5_. No statistically significant trends were observed for breast or prostate cancer incidence and mortality rates [per 1 μg/m^3^ PM_2.5_: breast cancer incidence 0.43 (95% CI −1.25, 2.12, p-value 0.61); breast cancer mortality −0.14 (95% CI −0.61, 0.33, p-value 0.57); prostate cancer incidence 1.80 (95% CI −0.64, 4.25, p-value 0.15); prostate cancer mortality 0.21 (95% CI −0.51, 0.93, p-value 0.57)].

When adjusting for neighborhood SES and prevalence of cigarette smoking, the associations between PM_2.5_ concentration and lung cancer incidence and mortality rates increased slightly. The adjusted slopes were 1.35 (95% CI 0.36, 2.35, p-value 0.01) per 1 μg/m^3^ PM_2.5_ for lung cancer incidence and 0.96 (95% CI 0.34, 1.59, p-value < 0.01) per 1 μg/m^3^ PM_2.5_ for lung cancer mortality. The R^2^ value, while still low, increased from 0.05 to 0.18 for lung cancer mortality. The R^2^ value for lung cancer incidence increased slightly from 0.07 to 0.09.

## 4. Discussion

The associations between PM_2.5_-concentration and lung cancer incidence and mortality rates provide limited evidence that PM_2.5_ exposure potentially contributes to lung cancer incidence and are consistent with previous studies that have reported an association between PM_2.5_ exposure and lung cancer mortality. A similar ecologic study performed using data from 15 countries in Europe reported a correlation between PM_2.5_ and lung cancer mortality among men but not women [8]. Recent work on the American Cancer Society cohort has demonstrated a positive association between PM_2.5_ and lung cancer mortality [2,3,6]. In addition, the Six Cities Study, which included follow-up from 1974–1998, reported a relative risk for lung cancer mortality of 1.27 (95% CI 0.96, 1.69) per 10 μg/m^3^ increase of PM_2.5_ [4]. However, other large studies (e.g., NLCS) have not detected an association between PM_2.5_ and lung cancer mortality [1], and some studies have found associations among only men or women [5,7]. The only previous study assessing the association between PM_2.5_ exposure and lung cancer incidence found no evidence of an association [1] while the studies of PM_10_ and lung cancer incidence have reported mixed results [9,10]. However, the results presented above support an association with lung cancer incidence.

The low R^2^ values represent the multifaceted factors that contribute to lung cancer incidence and mortality; just like many exposures, PM_2.5_ is not likely to be the sole etiologic agent responsible for increased lung cancer rates or deaths. The high variability demonstrates that tobacco smoking and other factors are contributing to lung cancer incidence and mortality rates. In addition, the smaller than expected change in R^2^ values after inclusion of variables for SES and smoking may indicate that there is still variability and confounding present in the association due to these factors.

This study is an important contribution to the limited literature available on the association between PM_2.5_ concentration and lung cancer incidence; our results suggest that further study of PM_2.5_ and lung cancer may be warranted. A strength of the study is the ability to include areas in North Carolina that are often excluded due to lack of monitoring data. This was possible because the PM_2.5_ air data was based on both monitoring and model data. In addition, this analysis included both lung cancer incidence and mortality, whereas many studies focus solely on lung cancer mortality, as rates of lung cancer incidence and mortality are often similar. However, in North Carolina the rates for the years included in this study are slightly different (72.9 per 100,000 *versus* 61.3 per 100,000), which indicates that there is some difference between incidence and mortality. The results of this study show higher lung cancer incidence rates compared with mortality rates in North Carolina and also demonstrated a steeper slope for PM_2.5_ and lung cancer incidence rates than for mortality rates. This study also provides a validation of the association observed between PM_2.5_ and lung cancer by assessing the association between PM_2.5_ concentration and breast and prostate cancers, two cancers so far unrelated to air pollution, and demonstrating no association in this ecologic analysis. This study is however limited by its ecologic nature. Assessment was performed at the county-level and the time periods utilized do not account for the latency period associated with lung cancer. However, due to the nature of PM_2.5_ as a regional pollutant, we expect that the counties that had higher concentrations of PM_2.5_ 20 or 30 years ago will be the same as those with higher PM_2.5_ concentrations during the period of our study. In other words, there is no reason for us to anticipate that the approximate rank order of the counties (by PM_2.5_ concentration) would have changed during the latency period of lung cancer, even though we recognize that, overall, concentrations of PM_2.5_ have decreased during this period. Because of the regional nature of PM_2.5_, we have assumed that this decrease was uniform across the counties. Similar assumptions may be made for county-level smoking rates. If these assumptions are incorrect, we would expect that it would be more difficult to identify an association between PM_2.5_ and lung cancer due to the addition of exposure measurement error; the fact that we observed a small, but statistically significant effect for PM_2.5_ and lung cancer, but not for breast or prostate cancer, leads us to believe that these assumptions hold. Another limitation is the lack of data on specific components of PM_2.5_, which could vary from county to county. PM_2.5_ concentrations were used in the analyses but specific components of PM_2.5_ might be more influential in lung cancer incidence and mortality. Nevertheless, data on PM_2.5_ components were not available. In addition, smoking rates were not available for each individual county and many of the counties’ rates were grouped together, resulting in no variance of smoking rates among those groups of counties. If the cigarette smoking rates did vary between those counties, this was not adjusted for in the above analyses and the true smoking prevalence may have been inadequately captured by the variable that was utilized. Finally, other factors besides smoking and SES (such as radon and asbestos exposure) may have played a role in lung cancer incidence and mortality rates and confounded the association. We were unable to account for these additional factors in this analysis.

In sum, few studies have explored the potential relationship between PM_2.5_ exposure and lung cancer incidence. Using an ecologic study design, we examined the county-level associations between modeled PM_2.5_ concentrations and lung cancer incidence and mortality in North Carolina. Although variability is high, which partly reflects the fact that there are other etiologic agents that influence lung cancer incidence and mortality besides PM_2.5_, there is a positive trend observed between PM_2.5_ concentration and lung cancer incidence and mortality rates. This provides evidence to suggest that PM_2.5_ concentrations are associated with lung cancer incidence and mortality rates. Future research at the individual-level, as opposed to an ecologic approach, will be important in understanding the role PM has on both lung cancer incidence and mortality as compared to and in interactions with tobacco smoking and other etiologic agents.
